# Applying the Concept of Peptide Uniqueness to Anti-Polio Vaccination

**DOI:** 10.1155/2015/541282

**Published:** 2015-10-19

**Authors:** Darja Kanduc, Candida Fasano, Giovanni Capone, Antonella Pesce Delfino, Michele Calabrò, Lorenzo Polimeno

**Affiliations:** ^1^Department of Biosciences, Biotechnologies, and Biopharmaceutics, University of Bari, 70126 Bari, Italy; ^2^Department of Emergency and Organ Transplantation (DETO), University of Bari, 70124 Bari, Italy

## Abstract

*Background*. Although rare, adverse events may associate with anti-poliovirus vaccination thus possibly hampering global polio eradication worldwide. *Objective*. To design peptide-based anti-polio vaccines exempt from potential cross-reactivity risks and possibly able to reduce rare potential adverse events such as the postvaccine paralytic poliomyelitis due to the tendency of the poliovirus genome to mutate. *Methods*. Proteins from poliovirus type 1, strain Mahoney, were analyzed for amino acid sequence identity to the human proteome at the pentapeptide level, searching for sequences that (1) have zero percent of identity to human proteins, (2) are potentially endowed with an immunologic potential, and (3) are highly conserved among poliovirus strains. *Results*. Sequence analyses produced a set of consensus epitopic peptides potentially able to generate specific anti-polio immune responses exempt from cross-reactivity with the human host. *Conclusion*. Peptide sequences unique to poliovirus proteins and conserved among polio strains might help formulate a specific and universal anti-polio vaccine able to react with multiple viral strains and exempt from the burden of possible cross-reactions with human proteins. As an additional advantage, using a peptide-based vaccine instead of current anti-polio DNA vaccines would eliminate the rare post-polio poliomyelitis cases and other disabling symptoms that may appear following vaccination.

## 1. Introduction

Vaccine-associated paralytic poliomyelitis (VAPP) [1] is the consequence of the replication of vaccine-derived polioviruses (VDPVs) that originate by genetic mutations from the strain contained in the oral polio vaccine (OPV). In fact, the poliovirus (PV) genetic mutability [2] appears to be the molecular basis of VAPP, which throws a shadow over the great success represented by vaccination in fighting PV infection [3, 4].

Currently, two new monovalent OPVs [5] and the change of the schedule from OPV to the exclusive use of inactivated polio vaccine (IPV) [6] represent options to interrupt PV transmission. However, it has been observed that reduction of exposure to a live attenuated virus such as that contained in OPV will inevitably lead to a decrease in herd immunity to a live microorganism and to natural boosters [7]. Such considerations, along with recent PV infection outbreaks, further complicate the issue of polio eradication [8].

In this scenario, new vaccine formulations and renewed research efforts might help to specifically fight PV infection. Recently, we analyzed the peptide overlap between PV1, strain Mahoney, and the human proteome and described a high extent of peptide sharing involving human proteins linked to fundamental cellular functions [9]. These data appeared to be of interest also because PV has been studied mainly at the nucleotide level [10], and, in general, there is a lack of knowledge of the interaction(s) between PV and the human host at the peptide/protein level. Actually, phenetic analyses of PV might help define peptide-based therapeutic approaches against PV infection, given that, for example,

- one single amino acid (aa) change, that is, His to Tyr at aa position 142 of virion protein 2 (VP2) or Val to Ile at aa position 160 of virion protein 1 (VP1), on the capsid surface of PV1 Sabin allows the establishment of persistent infections in HEp-2c cell cultures [11];
- replacement of the Ala residue with Asp at aa position 3 is linked to 50% loss of virion protein 4 (VP4) precursor myristoylation and severe reduction in specific infectivity [12];
- the 25th aa, Ile, of PV 2C protein interacts with human reticulon 3, a protein involved in viral replication and/or pathogenesis [13].

Moreover, since the 1980s we have known that small aa groupings can play a critical role in neutralizing PV. For example, the discontinuous E^4^T^7^T/S^8^R^9^ tetrapeptide, which is present in PV VP1, is crucial for neutralizing PV3 by anti-PV3 25-1-14 monoclonal antibody (mAb) [14]. Additional examples of short immune determinants in the immune response against PV are the following ones:

- A linear heptapeptide (DNNQTSP) is an Ab-binding site mapped to aa residues 164–170 of VP2 [15].
- Short synthetic peptides (e.g., DNTVRET, RSRSES, RSRSESSIESF, and STTNKDK) from PV VP1 prime the immune system of rabbits for a long-lasting, virus-neutralizing IgG Ab response following a single inoculation of intact virus [16].
- The Immune Epitope Database (IEDB; http://www.immuneepitope.org) [17] includes the continuous linear pentapeptide STTNK (IEDB ID: 61944) [18, 19] and the discontinuous pentapeptide T^141^E^143^S^312^E^315^P^417^ (IEDB ID: 91064) [20] as PV-derived epitopes.

Following the mathematical quantification of pentapeptide sharing between PV1 and human proteins, we reported that 2,040 out of the 2,204 pentamers composing the PV1 proteome are shared with the human proteome for a total of 18,223 matches, including multiple occurrences [9]. In general, a vast peptide sharing with human proteins is a characteristic of viral proteomes [21]. This peptide commonality suggests the existence of common evolutionary links between entities widely different as viruses and* Homo sapiens* and, in addition, indicates that potential cross-reactivity may affect antivirus vaccine formulations [22] and serological analyses [23]. Hence, it is reasonable to postulate that vaccines based on peptides unique to a virus and absent in the host proteome would guarantee high specificity and, at the same time, eliminate potential cross-reactivity.

Pursuing the objectives of overcoming the difficulties posed by the PV tendency to mutate and eliminating the viral reactivation-related VAPP in order to contribute to the global eradication of poliomyelitis, here we examine PV1 Mahoney polyprotein primary sequence and describe a set of pentapeptides uniquely owned by PV1, endowed with immunologic potential, and conserved among 43 PV strains. We propose that this set of pentapeptides might be used in preclinical and* in vivo* protocols with the ultimate aim of formulating effective, safe, and universal anti-PV vaccines.

## 2. Methods

The primary aa sequence of human PV1 polyproteins, strain Mahoney (NCBI taxonomic identifier: 12081; Swiss-Prot/UniProtKB entry: P03300), consisting of 11 viral proteins, 2,209 aa long [25] (further details at http://www.uniprot.org/uniprot/P03300), was analyzed for aa sequence similarity to the human proteome at the pentapeptide level. In brief, the viral polyprotein was dissected into 5-mers sequentially overlapped by four residues: MGAQV, GAQVS, AQVSS, QVSSQ, VSSQK, and so forth; then, each viral pentapeptide was used as a probe to scan the human proteome for instances of the same pentapeptide, as already described [9]. The similarity analysis used the Protein Information Resource (PIR) peptide match program (http://pir.georgetown.edu/) [26].

PV-derived epitopes were retrieved from the Immune Epitope Database (IEDB; http://www.immuneepitope.org) [17]. Only PV epitopes that had been experimentally validated in the human host were considered in this study.

Consensus peptide sequences were defined by ClustalW multialignment analysis (http://www.uniprot.org/program/?query=clustalw&sort=score) [24] of sequences from 43 PV strains retrieved from UniProt database (http://www.uniprot.org/) on the basis of the following characteristics: (1) described in scientific literature; (2) corresponding to the entire PV polyprotein; (3) derived from PV1 and PV3; (4) derived from PV variants isolated from VAPP or acute flaccid paralysis (AFP) patients or from immunocompromised patients with residual paralysis. Description and references of the 43 PV sequences used for multialignment analysis are reported in detail at http://www.uniprot.org/; the relative Swiss-Prot/UniProtKB entries are P03300, P03301, P03302, P06209, Q9Q281, Q9Q280, Q71AZ9, Q5TLH5, D1YSI9, D1YSJ1, D1YSJ2, D1YSJ3, D1YSJ4, D1YSJ5, D1YSJ6, D1YSJ7, D1YSJ9, D1YSK1, D1YSK2, D1YSK3, D1YSK4, D1YSK5, D1YSK6, D1YSK7, D1YSK8, D1YSK9, D1YSL0, D1YSL1, D1YSL2, D1YSL3, D1YSL4, D1YSL5, D2X673, D8L541, B4YUL3, B4YUL4, Q84792, C5HJY2, C5HJY3, D1GE40, D2E679, D1GE41, and D2XUS9.

## 3. Results and Discussion

### 3.1. Identification of Pentapeptides Unique to PV Type 1, Strain Mahoney

Using the procedure described under Section 2, we searched the human PV1, strain Mahoney, primary sequence for pentapeptides not shared with human proteins. We used pentapeptides as probes since a pentapeptide is a minimal functional unit in immunology [27], thus representing an appropriate length unit in measuring the qualitative/quantitative parameters of immunological phenomena [28].

Table 1 reports the pentapeptide platform that characterizes PV1 Mahoney polyprotein when compared to the* Homo sapiens* proteome. We find that 164 pentapeptides are unique to the viral polyprotein and absent in human proteins. In a few instances, viral 5-mers consecutively overlap (Table 1, pentapeptides in bold), thus forming 6-, 7-, and 8-mer stretches (e.g., PV_163–169_QNMYYHY, PV_446–453_NYYTHWAG, PV_783–789_AYSHFYD, PV_1496–1498_KGWIVNI, PV_1594–1600_GIHDNVA, PV_1699–1706_RTLMYNFP, and PV_1750–1756_EIQWMRP).

### 3.2. Analysis of the Immunologic Potential of Peptides Unique to PV

Next, the pentapeptides described in Table 1 were analyzed for their immunologic potential as follows. PV-derived epitopes were retrieved from IEDB, and the epitopes that had been experimentally validated in the human host were analyzed for the presence of pentapeptides unique to PV1 (see Table 1).

As reported in Table 2, the search through IEDB produced a final list of 78 viral epitopes derived from PV1 Mahoney, PV3 Sabin, and PV3 (P3/LEON/37 and P3/LEON 12A (1)B). Epitopes derived from PV2 strains were not considered since only data from immunoassays in mice, rats, and/or rabbits were available in IEDB for PV2 strains at the time of this study. Following sequence analysis, it was found that 20 of the 78 epitopes (i.e., IEDB IDs: 31814, 48785, 58511, 59797, 71769, 79272, 79480, 99910, 100138, 100244, 100349, 100382, 100536, 100576, 100631, 100667, 100672, 146181, 146248, and 146390; see IEDB IDs in bold in Table 2) have a total of 12 viral pentapeptides that are absent in the human proteome (pentapeptides in capital letters in Table 2). That is, a first conclusion from Table 2 is that 12 out of the 164 pentapeptides unique to PV1, strain Mahoney, are part of 20 PV epitopic sequences endowed with an immunologic potential in the human host.

Moreover, it can be seen that the 12 unique PV1 pentapeptides are not distributed at random among the PV-derived epitopes. For example, three unique pentapeptides (KWDDY, WDDYT, and YTWQT) overlap each other in the epitope IEDB ID 48785, sequence ppgapvpeKWDDYTWQTssnp (with unique overlapping pentapeptides in capital); the heptapeptide AYSHFYD, formed by three overlapping unique pentapeptides shifted by one residue, characterizes four PV-derived epitopes (IEDB IDs: 59797, 99910, 100244, and 100672); the hexapeptide SMMATG formed by two overlapped 5-mers is present in three epitopes (IEDB IDs: 146181, 146248, and 146390).

Theoretically, vaccines based on such PV epitopic peptides (i.e., KWDDYTWQT, AYSHFYD, and SMMATG) might evoke highly specific anti-PV immune responses exempt of possible collateral cross-reactions in the human host.

### 3.3. Identification of Consensus Pentapeptides Unique to PVs and Endowed with Immunologic Potential

We reasoned that using epitopic peptides unique to the virus and conserved among PV strains might help develop a global anti-PV peptide-based vaccination protocol. Such an approach would be of special importance in providing an effective and wide coverage to the human population worldwide, thus allowing reaching the goal of PV eradication [29, 30]. To this aim, we searched for unique conserved sequences by analysing a set of 43 PV polyproteins selected as described under Section 2 and comprehending also PV variants isolated from faeces of VAPP or AFP or from immunocompromised patient(s) with residual paralysis. The 43 PV polyprotein sequences were aligned using multialignment ClustalW program [24] and the peptide sequences present in PV-derived epitopes (see Table 2) and absent in the human proteome were localized.

Table 3 shows that seven potentially immunogenic peptides (in the order PV_465–469_MMATG, PV_708–712_RFDME, PV_752–756_DYTWQT, PV_763–767_FYTYG, PV_783–789_AYSHFYD, PV_835–839_KIRVY, and PV_847–851_VWCPR), derived from epitopes corresponding to (or formed of) pentapeptides unique to PV1 (see Tables 1 and 2), have 100% conservation among the 43 PV strains/variants under analysis. The seven potentially immunogenic unique peptide sequences had the same level of conservativeness in PV2 derived strains (data not shown).

## 4. Conclusion

Anti-PV immunization has been one of the major public health measures of the last century. International campaigns to eliminate polio reduced the incidence of this disease in the world. However, problematic issues remain, such as the tendency of the PV genome to mutate, the potential risk to develop postvaccine paralytic poliomyelitis, and the difficulty to completely eradicate PV infection in the world. In fact, according to the World Health Organization, in 2012, still three countries in the world remain polio-endemic: Nigeria, Pakistan, and Afghanistan [31].

The present data propose the concept of sequence uniqueness as a tool to define specific immunotherapies exempt of collateral effects [22] and describe a methodology to identify PV peptides that have zero percent identity to human proteins, are endowed with an immunologic potential, and are highly conserved among PV strains (e.g., PV_465–469_MMATG, PV_708–712_RFDME, PV_752–756_DYTWQT, PV_763–767_FYTYG, PV_783–789_AYSHFYD, PV_835–839_KIRVY, and PV_847–851_VWCPR; see Table 3). Importantly, polio peptide sequences alternate through the human proteome with a frequency versus rarity pattern that characterizes other pathogens too [32–35].

Theoretically, such viral consensus epitopic peptides appear to be ideal tools to generate anti-PV immune responses promising of high specificity, thus avoiding serological cross-reactivity between human polyomaviruses [23], as well as possible cross-reactions with the human host [22]. As an example, a construct composed of the coding frames corresponding to the immunogenic consensus sequences described above (Table 2) might determine a specific anti-PV immune response and, at the same time, by being based on peptides, might eliminate the issues inherent to the tendency of the PV genome to mutate (i.e., VAPP). Such viral peptide sequences might also be used in passive anti-PV immunotherapies, that is, to produce specific antibodies capable of reacting with intact viral protein antigens.

Actually, the present report is intended to represent a first approach to preclinical and animal studies. As a matter of fact, the solidity of a large body of theoretical and* in silico* data is the mandatory basis to design* in vivo* experimentation and validation protocols especially when considering that (i) although monkeys can be experimentally infected, humans are the only known natural hosts of poliovirus; (ii) small animal models for testing polio pathogenesis mainly relate to transgenic mice to express a human receptor to poliovirus [36]; and, moreover, (iii) current laws on* in vivo* experimentation are increasingly restrictive.

## Figures and Tables

**Table 1:** Pentapeptides unique to PV type 1, strain Mahoney, and absent in the human proteome.

Pos^a^	Sequence^b,c^	Pos^a^	Sequence^b,c^	Pos^a^	Sequence^b,c^	Pos^a^	Sequence^b,c^	Pos^a^	Sequence^b,c^	Pos^a^	Sequence^b,c^
11	GAHEN	393	MIPLN	746	PAKWD	969	YYPAR	1399	CHQPA	1750	**EIQWM**
29	TINYY	403	KNTMD	748	KWDDY	974	YQSHI	1403	ANFKR	1751	**IQWMR**
72	NIEAC	404	NTMDM	749	WDDYT	996	CHHGV	1413	CGKAI	1752	**QWMRP**
73	IEACG	408	MYRVQ	752	YTWQT	1014	FADIR	1418	QLMDK	1762	YPIIN
104	YGRWP	414	NDNPH	763	FYTYG	1021	YAYEE	1437	IVNER	1810	YVGNK
106	RWPEY	436	LSHTM	783	**AYSHF**	1084	ITRNY	1458	PIQYK	1840	TEQMC
107	WPEYL	446	**NYYTH**	784	**YSHFY**	1105	VSPWQ	1475	ECIND	1849	MYGTD
130	CRFYT	447	**YYTHW**	785	**SHFYD**	1136	TEACN	1489	VRNYC	1933	VAMRM
145	RGWWW	448	**YTHWA**	835	KIRVY	1139	CNAAK	1490	RNYCE	1935	MRMAF
146	GWWWK	449	**THWAG**	841	KPKHI	1188	STIHQ	1496	**KGWIV**	1946	FHKNP
148	WWKLP	464	SMMAT	847	VWCPR	1190	IHQSC	1497	**GWIVN**	1947	HKNPG
149	WKLPD	465	MMATG	857	AYYGP	1204	FNNVR	1498	**WIVNI**	1966	WSKIP
163	**QNMYY**	494	HVIWD	863	VDYKD	1205	NNVRW	1513	NRAMT	1979	AFDYT
164	**NMYYH**	495	VIWDI	880	TYGFG	1209	WLSIQ	1532	VYVMY	1983	TGYDA
165	**MYYHY**	497	WDIGL	881	YGFGH	1231	LEHTI	1534	VMYKL	1993	WFEAL
179	VQCNA	507	MVVPW	883	FGHQN	1291	FDGYK	1536	YKLFA	2028	YCVKG
199	MCLAG	510	PWISN	884	GHQNK	1311	GADMK	1588	GEFTM	2070	KMIAY
242	RFCPV	518	RQTTN	894	GYKIC	1314	MKLFC	1592	MLGIH	2073	AYGDD
244	CPVDY	534	FYQTR	895	YKICN	1326	FIPPM	1594	**GIHDN**	2115	TWENV
271	TNNCA	556	SACND	897	ICNYH	1340	FTSNY	1595	**IHDNV**	2131	KYPFL
292	KHNNW	557	ACNDF	912	VSTMW	1341	TSNYV	1596	**HDNVA**	2148	SIRWT
293	HNNWG	558	CNDFS	914	TMWDR	1368	RFAFD	1660	TETND	2158	TQDHV
295	NWGIA	568	DTTHI	934	ARCNC	1369	FAFDM	1677	MFVPV	2168	LAWHN
323	PMCC	569	TTHIG	942	VYYCE	1371	FDMDI	1699	**RTLMY**	2201	LYRRW
324	PMCCE	588	MIDNT	948	RRKYY	1388	DMTMA	1700	**TLMYN**		
326	CCEFN	665	CVSII	959	PTFQY	1389	MTMAT	1701	**LMYNF**		
327	CEFNG	708	RFDME	961	FQYME	1394	EMCKN	1702	**MYNFP**		
391	DTMIP	735	QIMYV	962	QYMEA	1398	NCHQP	1742	SYFTQ		

^a^aa position along the human PV type 1, strain Mahoney, primary sequence.

^b^aa sequences given in one-letter code.

^c^Consecutively overlapping pentapeptides forming 7- and 8-mer stretches unique to PV are given in bold.

**Table 2:** Twelve pentapeptides unique to PV1, Mahoney strain, and absent in the human proteome are distributed among twenty PV-derived epitopes recognized by human sera and/or T cells.

IEDB ID^a^	Epitope sequence^b,c^	PV antigen	PV strain	Immune context
30661	kevpaltavetgat	VP1	PV3 Sabin	B
**31814**	**klefftysRFDMEltfvvtan**	VP1	PV1 Mahoney	T
46859	paltavetgatnpl	VP1	PV1 Mahoney	B-T
**48785**	**ppgapvpeKWDDYTWQTssnp**	VP1	PV1 Mahoney	T
55952	rsrsessiesf	VP1	PV1 Mahoney	B
**58511**	**siFYTYGtaparisvpyvgi**	VP1	PV1 Mahoney	T
**59797**	**snAYSHFYDgfskvplkdqs**	VP1	PV1 Mahoney	T
66978	tvdnsasttnkdklfavwk	Polyprotein	PV1 Mahoney	T
**71769**	**vvndhnptkvtsKIRVYlkp**	Polyprotein	PV1 Mahoney	T
79155	altlslpkqqdslpdtka	Polyprotein	PV3 (P3/LEON/37 and P3/LEON 12A (1)B)	T
79160	atnplapsdtvqtrhvvq	Polyprotein	PV3 (P3/LEON/37 and P3/LEON 12A (1)B)	T
79186	dneqpttraqklfam	Polyprotein	PV3 (P3/LEON/37 and P3/LEON 12A (1)B)	T
79269	kevpaltavetgatnpla	Polyprotein	PV3 (P3/LEON/37 and P3/LEON 12A (1)B)	T
**79272**	**khvrVWCPRppravpyyg**	Polyprotein	PV3 (P3/LEON/37 and P3/LEON 12A (1)B)	T
79318	nghalnqvyqimyippga	Polyprotein	PV3 (P3/LEON/37 and P3/LEON 12A (1)B)	T
79350	qklfamwritykdtv	Polyprotein	PV3 (P3/LEON/37 and P3/LEON 12A (1)B)	T
79354	qpttraqklfamwri	Polyprotein	PV3 (P3/LEON/37 and P3/LEON 12A (1)B)	T
79433	traqklfamwrityk	Polyprotein	PV3 (P3/LEON/37 and P3/LEON 12A (1)B)	T
79434	traqklfamwritykdtv	Polyprotein	PV3 (P3/LEON/37 and P3/LEON 12A (1)B)	T
79435	trhvvqrrsrsestiesf	Polyprotein	PV3 (P3/LEON/37 and P3/LEON 12A (1)B)	T
79436	tskvriymkpkhvrvw	Polyprotein	PV3 (P3/LEON/37 and P3/LEON 12A (1)B)	T
79443	vaiievdneqpttraqkl	Polyprotein	PV3 (P3/LEON/37 and P3/LEON 12A (1)B)	T
79461	vrvvndhnptkvtskvri	Polyprotein	PV3 (P3/LEON/37 and P3/LEON 12A (1)B)	T
**79480**	**yippgaptpksWDDYTwq**	Polyprotein	PV3 (P3/LEON/37 and P3/LEON 12A (1)B)	T
80446	argacvtimtvdnpa	VP1	PV1 Mahoney	B
81394	easgpthskeipalt	VP1	PV1 Mahoney	B
82831	gpthskeipaltave	VP1	PV1 Mahoney	B
83234	hskeipaltavetga	VP1	PV1 Mahoney	B
88446	sdtvqtrhvvqhrsr	VP1	PV1 Mahoney	B
88495	sessiesffargacv	VP1	PV1 Mahoney	B
99863	aaparisvpyvgla	Polyprotein	PV3 Sabin	B
99886	ahskevpaltavet	Polyprotein	PV3 Sabin	B
99901	arisvpyvglanay	Polyprotein	PV3 Sabin	B
**99910**	**AYSHFYDgfakvpl**	Polyprotein	PV3 Sabin	B
99933	dfgvlavrvvndhn	Polyprotein	PV3 Sabin	B
99963	dtvqlrrkleffty	Polyprotein	PV3 Sabin	B
100029	famwritykdtvql	Polyprotein	PV3 Sabin	B
100101	hfydgfakvplktd	Polyprotein	PV3 Sabin	B
100117	hnptkvtskvriym	Polyprotein	PV3 Sabin	B
**100138**	**iFYTYGaaparisv**	Polyprotein	PV3 Sabin	B
**100244**	**lanAYSHFYDgfak**	Polyprotein	PV3 Sabin	B
**100349**	**npsiFYTYGaapar**	Polyprotein	PV3 Sabin	B
**100382**	**pkhvrVWCPRppra**	Polyprotein	PV3 Sabin	B
100391	psdtvqtrhvvqrr	Polyprotein	PV3 Sabin	B
100425	qklfamwritykdt	Polyprotein	PV3 Sabin	B
100430	qlrrklefftysrf	Polyprotein	PV3 Sabin	B
100439	qpttraqklfamwr	Polyprotein	PV3 Sabin	B
100482	rppravpyygpgvd	Polyprotein	PV3 Sabin	B
100492	samtvddfgvlavr	Polyprotein	PV3 Sabin	B
100504	sevaqgaltlslpk	Polyprotein	PV3 Sabin	B
**100536**	**svpyvglanAYSHF**	Polyprotein	PV3 Sabin	B
100559	tkvtskvriymkpk	Polyprotein	PV3 Sabin	B
100573	traqklfamwrity	Polyprotein	PV3 Sabin	B
100575	tskvriymkpkhvr	Polyprotein	PV3 Sabin	B
**100576**	**tssnpsiFYTYGaa**	Polyprotein	PV3 Sabin	B
100580	tvddfgvlavrvvn	Polyprotein	PV3 Sabin	B
100583	tvqtrhvvqrrsrs	Polyprotein	PV3 Sabin	B
100585	twqtssnpsifyty	Polyprotein	PV3 Sabin	B
100586	tygaaparisvpyv	Polyprotein	PV3 Sabin	B
100587	tykdtvqlrrklef	Polyprotein	PV3 Sabin	B
100613	vlavrvvndhnptk	Polyprotein	PV3 Sabin	B
100619	vndhnptkvtskvr	Polyprotein	PV3 Sabin	B
100628	vriymkpkhvrvwc	Polyprotein	PV3 Sabin	B
100630	vrvvndhnptkvts	Polyprotein	PV3 Sabin	B
**100631**	**vrVWCPRppravpy**	Polyprotein	PV3 Sabin	B
100638	wcprppravpyygp	Polyprotein	PV3 Sabin	B
100644	writykdtvqlrrk	Polyprotein	PV3 Sabin	B
**100667**	**ymkpkhvrVWCPRp**	Polyprotein	PV3 Sabin	B
**100672**	**yvglanAYSHFYDg**	Polyprotein	PV3 Sabin	B
146178	ayappgaqpptsrk	Polyprotein	PV3 Sabin	B
**146181**	**cgSMMATGkilvay**	Polyprotein	PV3 Sabin	B
**146248**	**flfcgSMMATGkil**	Polyprotein	PV3 Sabin	B
146311	hqgalgvfaipeyc	Polyprotein	PV3 Sabin	B
146333	ilvayappgaqppt	Polyprotein	PV3 Sabin	B
**146390**	**kftflfcgSMMATG**	Polyprotein	PV3 Sabin	B
146494	phqiinlrtnnsat	Polyprotein	PV3 Sabin	B
146496	ppgaqpptsrkeam	Polyprotein	PV3 Sabin	B
146516	ravpyygpgvdyrn	Polyprotein	PV3 Sabin	B

^a^PV-derived epitopes are listed according to increasing IEDB ID number. Further details and reference(s) on each IEDB ID are reported at http://www.immuneepitope.org [17].

^b^Only PV-derived epitopes that had been experimentally validated in the human host are reported.

^c^The twenty PV-derived epitopes (and related IDs) containing PV-pentapeptide(s) absent in the human proteome are in bold, with the pentapeptide(s) absent in the human proteome given in capital.

**Table 3:** Conservativeness of epitopic PV peptide regions among 43 PV strains and variants.

PV strain	UniProt/Swiss-Prot entry	aa Pos 464	aa Pos 708	aa Pos 748	aa Pos 763	aa Pos 783	aa Pos 835	aa Pos 847
PV1 Mahoney	P03300	FMMATG	RFDME	KWDDYTWQT	FYTYG	AYSHFYD	KIRVY	VWCPR
PV1 Sabin	P03301	SMMATG	RFDME	KWDDYTWQT	FYTYG	AYSHFYD	KIRVY	VWCPR
PV3 P3/Leon/37	P03302	SMMATG	RFDME	sWDDYTWQT	FYTYG	AYSHFYD	KvRiY	VWCPR
PV3 23127	P06209	SMMATG	RFDME	sWDDYTWQT	FYTYG	AYSHFYD	KvRvY	VWCPR
PV1 isolated	Q9Q281	SMMATG	RFDME	KWDDYTWQT	FYTYG	AYSHFYD	KvRVY	VWCPR
PV1 isolated	Q9Q280	SMMATG	RFDME	KWDDYTWQT	FYTYG	AYSHFYD	KIRVY	VWCPR
PV1 isolated	Q71AZ9	SMMATG	RFDME	KWDDYTWQT	FYTYG	AYSHFYD	KIRVY	VWCPR
PV1 isolated	Q5TLH5	SMMATG	RFDME	KWDDYTWQT	FYTYG	AYSHFYD	KIRVY	VWCPR
PV1-HAI01008C2	D1YSI9	SMMATG	RFDME	KWDDYTWQT	FYTYG	AYSHFYD	KIRVY	VWCPR
PV1-HAI01009	D1YSJ1	SMMATG	RFDME	KWDDYTWQT	FYTYG	AYSHFYD	KIRVY	VWCPR
PV1-HAI01008	D1YSJ2	SMMATG	RFDME	KWDDYTWQT	FYTYG	AYSHFYD	KIRVY	VWCPR
PV1-HAI01002	D1YSJ3	SMMATG	RFDME	KWDDYTWQT	FYTYG	AYSHFYD	KIRVY	VWCPR
PV1-HAI01001	D1YSJ4	SMMATG	RFDME	KWDDYTWQT	FYTYG	AYSHFYD	KIRVY	VWCPR
PV1-DOR01012	D1YSJ5	SMMATG	RFDME	KWDDYTWQT	FYTYG	AYSHFYD	KIRVY	VWCPR
PV1-DOR01002C	D1YSJ6	SMMATG	RFDME	KWDDYTWQT	FYTYG	AYSHFYD	KIRVY	VWCPR
PV1-DOR01002	D1YSJ7	SMMATG	RFDME	KWDDYTWQT	FYTYG	AYSHFYD	KIRVY	VWCPR
PV1-DOR01001C1	D1YSJ9	SMMATG	RFDME	KWDDYTWQT	FYTYG	AYSHFYD	KIRVY	VWCPR
PV1-DOR00042C2	D1YSK1	SMMATG	RFDME	KWDDYTWQT	FYTYG	AYSHFYD	KIRVY	VWCPR
PV1-DOR00042C1	D1YSK2	SMMATG	RFDME	KWDDYTWQT	FYTYG	AYSHFYD	KIRVY	VWCPR
PV1-DOR00042	D1YSK3	SMMATG	RFDME	KWDDYTWQT	FYTYG	AYSHFYD	KIRVY	VWCPR
PV1-DOR00041C3	D1YSK4	SMMATG	RFDME	KWDDYTWQT	FYTYG	AYSHFYD	KIRVY	VWCPR
PV1-DOR00041C2	D1YSK5	SMMATG	RFDME	KWDDYTWQT	FYTYG	AYSHFYD	KIRVY	VWCPR
PV1-DOR00044	D1YSK6	SMMATG	RFDME	KWDDYTWQT	FYTYG	AYSHFYD	KIRVY	VWCPR
PV1-DOR00028C	D1YSK7	SMMATG	RFDME	KWDDYTWQT	FYTYG	AYSHFYD	KIRVY	VWCPR
PV1-DOR00028	D1YSK8	SMMATG	RFDME	KWDDYTWQT	FYTYG	AYSHFYD	KIRVY	VWCPR
PV1-DOR00023C	D1YSK9	SMMATG	RFDME	KWDDYTWQT	FYTYG	AYSHFYD	KIRVY	VWCPR
PV1-DOR00025	D1YSL0	SMMATG	RFDME	KWDDYTWQT	FYTYG	AYSHFYD	KIRVY	VWCPR
PV1-DOOR24	D1YSL1	SMMATG	RFDME	KWDDYTWQT	FYTYG	AYSHFYD	KIRVY	VWCPR
PV1-DOR00015	D1YSL2	SMMATG	RFDME	KWDDYTWQT	FYTYG	AYSHFYD	KIRVY	VWCPR
PV1-DOR00016	D1YSL3	SMMATG	RFDME	KWDDYTWQT	FYTYG	AYSHFYD	KIRVY	VWCPR
PV1-HAI01013all	D1YSL4	SMMATG	RFDME	KWDDYTWQT	FYTYG	AYSHFYD	KIRVY	VWCPR
PV1-HAI01015	D1YSL5	SMMATG	RFDME	KWDDYTWQT	FYTYG	AYSHFYD	KIRVY	VWCPR
PV1-S302	D2X673	SMMATG	RFDME	KWDDYTWQT	FYTYG	AYSHFYD	KIRVY	VWCPR
CHN8184/GZ/CHN/2004	D8L541	SMMATG	RFDME	KWDDYTWQT	FYTYG	AYSHFYD	KIRVY	VWCPR
PV3 isolated	B4YUL3	SMMATG	RFDME	sWDDYTWQT	FYTYG	AYSHFYD	KvRVY	VWCPR
PV3 isolated	B4YUL4	SMMATG	RFDME	sWDDYTWQT	FYTYG	AYSHFYD	KvRVY	VWCPR
PV3 (vacc.StrainSabin3 (Leon 12a1b))	Q84792	SMMATG	RFDME	sWDDYTWQT	FYTYG	AYSHFYD	KvRiY	VWCPR
PV3-33239	C5HJY2	SMMATG	RFDME	sWDDYTWQT	FYTYG	AYSHFYD	KvRiY	VWCPR
PV3-31974	C5HJY3	SMMATG	RFDME	sWDDYTWQT	FYTYG	AYSHFYD	KvRiY	VWCPR
PV3-FIN84-60212	D1GE40	SMMATG	RFDME	sWDDYTWQT	FYTYG	AYSHFYD	KvRiY	VWCPR
PV3-SWI10947	D2E679	SMMATG	RFDME	sWDDYTWQT	FYTYG	AYSHFYD	KvRiY	VWCPR
PV3-FIN84-2493	D1GE41	SMMATG	RFDME	sWDDYTWQT	FYTYG	AYSHFYD	KvRiY	VWCPR
PV3-P3/Jinan/1/09	D2XUS9	SMMATG	RFDME	sWDDYTWQT	FYTYG	AYSHFYD	KvRiY	VWCPR

The 43 PV sequences were aligned using ClustalW multialignment program (http://www.uniprot.org/align/) [24]. The analyzed PV sequences and related references are described at http://www.uniprot.org/ and reported by Swiss-Prot/UniProtKB accession number. Peptide sequences present in PV-derived epitopes (see Table 2) and absent in the human proteome were localized along the 43 aligned PV sequences and analyzed for conservativeness. Peptide sequences are indicated by their position along the PV polyprotein. Mutated aa residues are in lower case.
